# Assessment of Hearing Loss in Two-Year Follow-up Study of Neonates with Congenital Cytomegalovirus Infection

**DOI:** 10.22037/ijcn.v16i2.30592

**Published:** 2022-03-14

**Authors:** Samileh NOORBAKHSH, Mohammad Taghi JOGHATAEI, Mohammad FARHADI, Faezeh HAGHIGHI, Hesamaldin EMAMJOMEH, Morteza HAGHIGHI HASANABAD

**Affiliations:** 1 Department of Pediatric Infectious Diseases, Iran University of Medical Sciences, Tehran, Iran; 2 Department of Neuroscience, Faculty of Advanced Technologies in Medicine, Iran University of Medical Sciences, Tehran, Iran; 3 Cellular and Molecular Research Center, Iran University of Medical Sciences, Tehran, Iran; 4 ENT and Head & Neck Research Center and Department, Iran University of Medical Sciences, Tehran, Iran; 5 Cellular and Molecular Research Center, Sabzevar University of Medical Sciences, Sabzevar, Iran; 6 Pediatric Infectious Diseases Research Center, Institute of Immunology and Infectious Diseases, Iran University of Medical Sciences, Tehran, Iran

**Keywords:** Hearing Loss, Neonates, Congenital Cytomegalovirus infection

## Abstract

**Objectives:**

Congenital Cytomegalovirus **(**cCMV) infection constitutes the main cause of sensory neural hearing loss (SNHL) worldwide. The rate of Cytomegalovirus** (**CMV)-induced SNHL is not well documented in developing countries, such as Iran. Therefore, this prospective follow-up study aimed to evaluate this rate among neonates with cCMV infection in Iran.

**Materials & Methods:**

Neonates with cCMV infection admitted to neonatal intensive care units and neonates with CMV infection identified in two other prospective screening studies in Tehran, Iran, were enrolled in this study. Audiological assessments, including otoacoustic emission and auditory brainstem response tests, were performed for all the cases. Antiviral therapy was administered for the newborns in case of having severe symptoms.

**Results:**

A total of 22 neonates with cCMV infection were entered into the study, of whom 8 and 14 subjects had symptomatic and asymptomatic cCMV infection, respectively. In total, 3 of 22 newborns had SNHL (13.6%; 95% CI: 2.8-39.8), 2 of 8 cases with symptomatic cCMV infection (25.0%; 95% CI: 3-90) and 1 of 14 cases with asymptomatic cCMV infection (7.1%; 95% CI: 0.1-39). No association was observed between SNHL and CMV-related risk factors in newborns.

**Conclusion:**

The findings of this study revealed that the rate of cCMV-induced SNHL is high among neonates born in Tehran. The severe sequelae of cCMV infection indicate the need for screening for CMV infection at birth to reduce the risk of CMV complications and the financial load of treatment imposed on healthcare and treatment systems in Iran.

## Introduction

Congenital Cytomegalovirus(cCMV) is the leading cause of congenital anomalies and neurological damage in children worldwide (1). The annual incidence of cCMV infection is estimated within the range of 2-22 per 1000 live births in the world (2). It is important to note that due to postnatal exposure to Cytomegalovirus (CMV), newborns might shed the virus in their urine or saliva after 21 days of birth. Therefore, the diagnosis of cCMV infection should be made within the first 3weeks of the neonate’s life (3).

Generally, more than 90% of newborns with cCMV infection are asymptomatic at birth, and of the remaining 10% infected neonates, numerous cases go unidentified due to the lack of specific symptoms or signs of CMV infection (4). Unfortunately, the complications of cCMV infection with continuing risk through adult life encompass lifelong disabilities, such as sensory neural hearing loss (SNHL) (i.e., hearing loss [HL] due to the auditory nerve and/or cochlear damage) and neurological impairment (5). It is estimated that about 25% of all cases with HL (in children up to 5years of age) are related to cCMV infection, less than one-third of which are recognized in newborn hearing screening due to the late onset of this sequela (6). 

Moreover, it is estimated that approximately 9.3-17% of neonates with cCMV infection will develop SNHL during childhood (7, 8). It is important to note that this viral infection is known as the leading non-genetic cause of SNHL worldwide. Given that, universal CMV screening and targeted CMV screening of neonates who failed in neonatal hearing screening tests are two approaches that gained more attention(9).

Based on published investigations, the awareness of the epidemiology of cCMV infection and its role as a cause of HL in neonates born to mothers with high CMV seroprevalence is of considerable importance. Recently, some studies have demonstrated that HL occurs at a similar range in neonates born to mothers with non primary CMV infection and women with primary CMV infection (10, 11). However, the rates of CMV-induced SNHL and related risk factors in populations with high maternal CMV seroprevalence, such as Iran, have not been well documented to date. Therefore, this prospective follow-up study aimed to evaluate the rates of SNHL and its associated factors in a population of newborns with cCMV infection born to mothers with near-universal CMV seroprevalence.

## Materials & Methods

In this prospective follow-up study, neonates with cCMV infection admitted to the neonatal intensive care units (NICUs) of university-affiliated hospitals in Tehran, Iran, and CMV-infected neonates identified in two prospective screening studies of 1398 neonates born in six cities of Tehran province (4/1174 and 11/224 asymptomatic and hospitalized neonates, respectively) were enrolled within April-September in 2017(12, 13).

The inclusion criterion for cases was the detection of CMV deoxyribonucleic acid(DNA) in the urine or dried blood spot (DBS) samples of neonates within the first 3 weeks of life (<21 days of birth). The exclusion criteria were defined as the inability of parents to sign an informed consent form, unwillingness to continue the study, and/or absence in scheduled follow-up visits twice.

This study was approved by the Ethics Committee affiliated with Iran University of Medical Sciences, Tehran, Iran, and the study followed the principles outlined in the declaration of Helsinki. Written informed consent was obtained from the parents of all infected neonates for participating in this study. The clinical characteristics and demographic data of neonates and related maternal factors were collected by questionnaires or documented from their medical records. Educational brochures containing data about CMV infection, transmission, and prevention were distributed between newborns’ parents.

Total DNA was isolated from the DBS samples in triplicate using heat shock assay according to a modified protocol(14). Briefly, the DBS samples were soaked in Minimum Essential Medium (Sigma-Aldrich Co., USA) and incubated at 4°C overnight and then heated by a thermocycler with a program at 55°C for 60 minutes, 100°C for 7minutes, and0°C for 2minutes (Eppendorf, Germany). Then, the DBS samples were centrifuged at 3320 × g for 15 minutes, and the supernatant was incubated at −80 °C overnight. Additionally, nucleic acids were extracted from urine specimens using a commercial extraction kit (Roche Diagnostics, Germany) and according to the manufacturer’s instructions. The presence of CMV DNA in the samples was tested using an in-house nested polymerase chain reaction(PCR) as described previously, and cCMV infection in newborns with positive results was confirmed via testing by a quantification PCR kit (13).

All neonates with cCMV infection were examined by two pediatricians and underwent a clinical evaluation, laboratory tests, ophthalmological examination, liver function test, and computed tomography (CT) scan of the brain, if necessary. Antiviral drugs were administered for the newborns in case of having severe symptoms and based on physician decisions.

The results of the hearing screening test of newborns consisting of transient otoacoustic emission (OAE) testing at the birth time were received from their parents and documented as “normal” or “fail” results. The infected neonates with impaired OAE were referred to an otolaryngologist in the Department of Head, Neck, and Ear Surgery in Hazrate Rasool hospital in Tehran for treatment. The otoscopic examination was performed for the infected neonates to diagnose any middle-ear disorders. Then, an auditory brainstem response (ABR) test using the threshold method was performed for all the cases with cCMV infection within 3 months of life and subsequently every 6 months up to the age of 24 months (15). The ABR test was conducted after the neonate’s sedation with chloral hydrate. A newborn with normal middle ear function was considered to have normal hearing when the click threshold was less than 30 dB in either ear. The HL in neonates was classified as mild, moderate, severe, and profound when the click threshold was within the ranges of31-45, 46-70, 71-90, and ≥91 dB, respectively (16).

The data were collected and entered into the datasheet and analyzed using MedCalc software for Windows (version 18.11; Ostend, Belgium). Univariate analyses were performed by Fisher’s exact test and Chi-square test to compare the clinical findings and demographic characteristics between neonates with and without SNHL. A p-value of less than 0.05 was considered statistically significant.

## Results

Of 23 eligible newborns with confirmed cCMV infection, 1caserelocated to another city and was excluded from the study. Moreover, 9 and 13 of 22 neonates were female and male, respectively. The mean age of neonates at the screening time was 16 days. The clinical symptoms and signs consistent with symptomatic cCMV infection were observed in eight neonates (at least one specific symptom) five of whom had multisystem diseases and underwent antiviral therapy (ganciclovir, 6mg/kg/ per 12 hours for 6 weeks), based on the pediatric decision. The remaining 14 neonates had no specific symptoms related to cCMV infection and were classified under asymptomatic CMV infection.

**Clinical Findings**

In this study, 14 of 22 infected neonates presented jaundice after birth, of whom 10 subjects did not require phototherapy; however, 4 subjects had severe jaundice and underwent phototherapy. The peripheral blood smear of seven neonates revealed few erythroblasts and pancytopenia. Germinal matrix hemorrhage was observed in the brain ultrasound of one infected newborn. In two neonates, the abdominal ultrasound scan revealed hepatomegaly and splenomegaly; nevertheless, the kidney size was normal. Respiratory distress syndrome was observed in one infected neonate; nonetheless, the chest X-ray was normal. The brain CT scan in one newborn revealed mild hydrocephaly with no signs of calcification. When this neonate was treated with ganciclovir, a significant reduction was observed in spleen size; however, no increase was observed in head circumference. In ophthalmologic assessment, retinal hemorrhage without the inflammation of systemic deficits was detected in one neonate. Additionally, chorioretinitis was reported in one infected newborn after 9 months of birth.

**Hearing Status of Neonates and Audiological Follow-up**

**Figure1:**
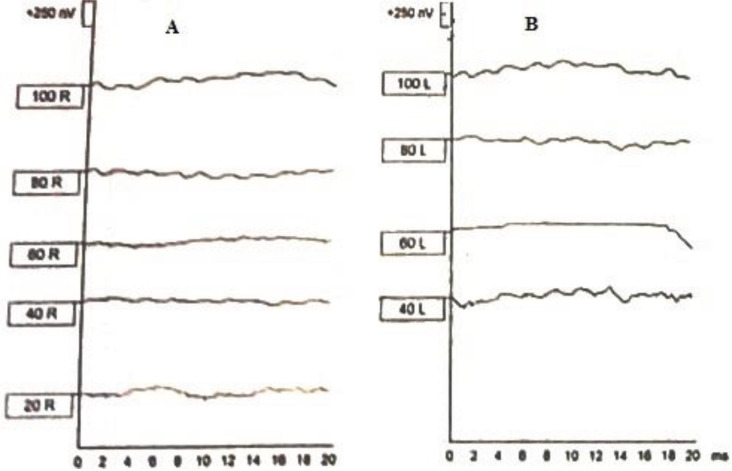
Result of ABR Test in a Neonate with Profound Bilateral Hearing Loss. A; Right Ear, B; Left Ear

All infected neonates completed a follow-up study for 24 months of age and underwent at least one OAE test and five ABR tests. Accordingly, 19out of 22 newborns with cCMV infection initially passed the OAE test as normal; nevertheless, the OAE test was abnormal for three subjects. One of the three newborns with failed OAE tests during initial hearing screening revealed normal response in subsequent assessments by OAE and ABR tests. On the other hand, a child with a normal OAE test at initial hearing screening developed SNHL during the follow-up period at 18 months of age (this neonate was born to a mother with primary CMV infection). Therefore, of 22 newborns undergoing audiological evaluation by the ABR test during 24 months, 3 subjects were demonstrated to have SNHL (13.6%; 95% CI: 2.8-39.8), 2 of 8 cases with symptomatic cCMV infection (25.0%; 95% CI: 3-90) and 1 of 14 cases with asymptomatic cCMV infection (7.1%; 95% CI: 0.1-39). Two of the three neonates had moderate unilateral HL (>45-70 dB), and the remaining neonate had a profound bilateral HL (>90dB). Figure 1 shows electrophysiological results of ABR test in a newborn with cCMV infection.

Table 1 shows the comparison of clinical findings and demographic characteristics of newborns with and without SNHL. No association was observed between SNHL with gender, maternal age, intrauterine growth restriction, jaundice, and preterm birth in newborns.

**Table 1:** Clinical remarks and demographic characteristics of neonates with cCMV infection according to SNHL

**Variable**	**Situation**	**SNHL in Neonates**		**P-value **
		**Positive** **(n=3)**	**Negative (n=19)**	
Intrauterine Growth Restriction (IUGR)	Yes	2 (28.5%)	5 (77.5%)	0.2
	No	1 (6.6%)	14 (93.4%)	
Jaundice	Yes	2 (14.2%)	12 (85.8%)	0.9
	No	1 (12.5%)	7 (87.5%)	
Preterm birth	Yes	2 (15.3%)	11 (84.7%)	0.7
	No	1 (11.1%)	8 (88.9%)	
Gender	Male	1 (7.6%)	12 (92.4%)	0.3
	Female	2 (22.2%)	7 (77.8%)	
Maternal age	≤27	2 (13.3%)	13 (86.7%)	0.9
	>27	1 (14.2%)	6 (85.8%)	

**Maternal Factors**

The results of CMV serologic status during the first trimester of pregnancy were available for 17 of 22 mothers with CMV infected neonates, of whom11 subjects were previously infected with CMV and classified as women with non primary CMV infection (based on the presence of CMV immunoglobulin G antibody in their serum samples) and 6 subjects were infected newly with CMV and classified as mothers with primary CMV infection. Table 2 shows the data on the association between HL in newborns and the type of CMV infection in their mothers. Additionally, the level of CMV awareness among neonates’ parents was very low, and none of them had heard about CMV before this project.

**Table 2:** Hearing loss in neonates according to the type of CMV infection in their mothers

**Outcome in Neonates**	**Situation**	**Type of CMV Infection in Mothers**		**P-value**
		**Primary (n=6)**	**Non-primary (n=11)**	
Hearing Loss	Yes	1	1	0.4
	No	5	10	

## Discussion

One of the paramount etiologies of pediatric HL is cCMV infection (16). According to the available data, the primary effects of the destruction of the nervous system, especially the occurrence of CMV-induced HL, often start by transferring the virus from the mother to the fetus during pregnancy, and the infection gradually manifests itself after birth (17). Therefore, due to the high prevalence and severity of the complications, it seems that screening for cCMV infection in newborns should be necessary.

To the best of our knowledge, this has been the first study to investigate the rate of SNHL among neonates with cCMV infection in Iran. The current study was conducted on newborns with cCMV infection identified through screening newborns within the first 3 weeks of life (8 infected neonates admitted to the NICUs and 11 and 4 cases hospitalized and asymptomatic, respectively, and entered from two other prospective screening studies). The obtained results revealed that the frequency of CMV-related SNHL in this region of Iran is about 13.6% (3 of 22 subjects). This result is consistent with those reported from studies performed in developed countries with moderate to low maternal CMV seroprevalence, in which 9.3-17% of neonates with cCMV infection are reported to have SNHL (7, 8). Additionally, according to the results of the aforementioned studies, the rates of SNHL in children with symptomatic and asymptomatic CMV infection are within the ranges of 22-41% and 6-16%, respectively. 

The above-mentioned data confirm the findings of this study in which 25% and 7.1% of CMV infected neonates with SNHL were among symptomatic and asymptomatic cases, respectively. Although this rate should be lower in Iran due to high maternal CMV seroprevalence, differences in study enrollment criteria between the present survey and the above-mentioned investigations conducted in developed countries with low CMV seroprevalence probably justify the present study’s findings. It is important to note that 8 of 22 neonates with cCMV infection in the current study were enrolled from newborns admitted to the NICU of the hospitals who generally have more chances to develop HL(18).

The present study’s findings demonstrated that the rate of CMV-induced SNHL in Tehran is about 2 per 22 cases. In addition, based on the previous data from Mashhad and Yazd located in the northeast and center of Iran, the incidence of HL in Iran is reported within the range of3.5-6.5 per 1000 live births(19, 20). Although one of three neonates with SNHL in the present study was normal during initial hearing screening and developed HL at 18 months of age, within 3-5% of HL in Iran is due to CMV, according to the aforementioned data. Considering the 0.5% birth prevalence rate of cCMV infection in Iran(21), SNHL due to cCMV infection would affect at least 5 neonates per 10,000 live births or 600 of the 1.200,000 Iranian newborns born annually. Moreover, 1/3 (200 neonates) with moderate SNHL had not been identified due to the late-onset HL and lack of any clinical symptoms related to CMV infection at birth; therefore, they would not have been identified during the initial hearing screening test if they were not screened for CMV and underwent HL follow-up.

In the present study, 4.5% of neonates who failed in initial hearing screening by the OAE test were infected with cCMV. This finding supports the findings of studies that indicated cCMV infection accounted for about 6% of neonates with HL (22). Other retrospective observational studies have shown that within5-8% of neonates who failed in hearing screening tests are estimated to be infected with CMV (23, 24).

In the current study, 9% and 16.6% of infected newborns with SNHL were born to mothers with non primary and primary CMV infection, respectively. This result is consistent with recently published reports of studies in which 7-10% and 11-15% of infected neonates born to mothers with non primary and primary CMV infection had SNHL, respectively(10, 11).

The data of the present study revealed that no parents in this study had previously heard about CMV or CMV-associated symptoms. Although an efficient vaccine for cCMV infection is not available at present, the risk of CMV infection among populations can be reduced by enhancing CMV awareness level about prevention-based strategies, such as further attention to hygiene practices and routine hand-washing with soap (25).

The current study’s results should be considered in light of some limitations. Firstly, due to the small sample size of 22 infected neonates with cCMV infection, the statistical data appear to be insufficient to properly calculate the SNHL associated risk factors. Secondly, the participants were mainly selected from the infected neonates who were hospitalized or from admitted neonates to NICUs that might affect the outcome analyses. Finally, the study site was located in one region; therefore, the findings might not be generalizable to all regions of Iran. 

## In Conclusion

The findings of this study demonstrate that cCMV infection is one of the most important infectious factors inducing SNHL in neonates, and neonates with symptomatic CMV infection are more prone to develop HL. The results also indicate that neonatal screening for cCMV infection at birth and further assessment of infected newborns even with normal hearing status is necessary to identify the late-onset HL in children and start antiviral therapy to prevent disease progression. Considering other serious consequences of cCMV infection in addition to HL, it would be of interest to clarify different effects of this infection on neonates and determine their incidence in this population; therefore, future screening tests can be programmed similar to those implemented for metabolic diseases.

## Author’s contribution

Samileh Noorbakhsh: Thesis Supervisor, designed the study, acquired and interpreted the data and critically revised the manuscript; Mohammad Taghi Joghataei: Thesis Supervisor, acquired and interpreted the data and critically revised the manuscript; Mohammad Farhadi: Thesis Supervisor, acquired and interpreted the data and monitored audiological assessments; Faezeh Haghighi: Collected and analyzed the data; Hesamaldin Emamjomeh: Carried out the audiological tests; Morteza Haghighi Hasanabad: Collected and tested the samples, and was involved in all the steps of the experiments, drafted and critically revised the study manuscript. All the authors have read and approved the final manuscript prior to submission.

## Conflict of interest

The authors declare that they have no conflict of interest.
